# Evidence for mirror systems in emotions

**DOI:** 10.1098/rstb.2009.0058

**Published:** 2009-08-27

**Authors:** J. A. C. J. Bastiaansen, M. Thioux, C. Keysers

**Affiliations:** 1 BCN NeuroImaging Center, University of Groningen, Antonius Deusinglaan 2, 9713 AW Groningen, The Netherlands; 2 Department of Neuroscience, University Medical Center Groningen, Antonius Deusinglaan 1, 9713 AW Groningen, The Netherlands; 3 Autism Team North Netherlands, Lentis, Hereweg 80, 9725 AG Groningen, The Netherlands

**Keywords:** emotions, sensations, simulation, mirror system, empathy, social cognition

## Abstract

Why do we feel tears well up when we see a loved one cry? Why do we wince when we see other people hurt themselves? This review addresses these questions from the perspective of embodied simulation: observing the actions and tactile sensations of others activates premotor, posterior parietal and somatosensory regions in the brain of the observer which are also active when performing similar movements and feeling similar sensations. We will show that seeing the emotions of others also recruits regions involved in experiencing similar emotions, although there does not seem to be a reliable mapping of particular emotions onto particular brain regions. Instead, emotion simulation seems to involve a mosaic of affective, motor and somatosensory components. The relative contributions of these components to a particular emotion and their interrelationship are largely unknown, although recent experimental evidence suggests that motor simulation may be a trigger for the simulation of associated feeling states. This mosaic of simulations may be necessary for generating the compelling insights we have into the feelings of others. Through their integration with, and modulation by, higher cognitive functions, they could be at the core of important social functions, including empathy, mind reading and social learning.

## 1. Introduction

Humans have an astonishing capacity to intuitively grasp the mental states of other individuals. If we see someone bite into his sandwich and show a horrified grimace, we do not have to chew over what happened to sense that he is not enjoying his meal. In fact, just the sight of his disgust might cause our own stomachs to turn and prevent us from eating our own sandwich. Although people's more subtle emotions can remain puzzling, we often have gut feelings of what is going on in other individuals. Various researchers have suggested under different designations that direct simulation (see glossary) of observed social events through mirror-like mechanisms are at the heart of this experiential understanding of others: the shared-manifold hypothesis (Gallese 2003); unmediated resonance model (Goldman & Sripada 2005); shared circuits (Keysers & Gazzola 2006); direct-matching hypothesis (Rizzolatti *et al*. 2001) and hot hypothesis (Wicker *et al*. 2003). The basic tenet of these models is that observation of an action in another individual directly triggers activation of matching neural substrates in the observer through which the action can be understood. While some researchers focus on the role of motor areas in social cognition (e.g. motor theory of social cognition, Jacob & Jeannerod 2005), others see embodied simulation as a general and basic endowment of our brain that involves a linkage between the first and third person experiences of actions, sensations and emotions (Keysers & Gazzola 2006).

### (a) Sharing actions in the premotor and parietal cortex

**Figure 1.**
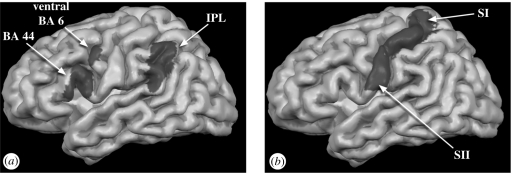
Anatomical locations of the motor and somatosensory components of simulation. (*a*) Lateral view of the human brain with the location of the ventral premotor cortex (BA6/BA44) and the inferior parietal lobule (IPL). (*b*) Lateral view showing the location of the primary and secondary somatosensory cortex (SI/SII).

Simulation theories were greatly stimulated by the study of action execution and action observation in monkeys. Two reciprocally connected areas, namely area F5 in the ventral premotor cortex and the parietal area PF, were found to contain individual neurons that respond both to the execution of hand-object interactions and the sight of similar actions (see Keysers & Perrett 2004; Rizzolatti & Craighero 2004 for reviews). Owing to their common role in first (I grasp) and third person (he grasps) perspectives, these neurons were named ‘mirror neurons’. Linking what the monkey sees people do to what it does itself might provide it with an intuitive insight into the actions of others. Given their properties, mirror neurons seem particularly well suited to providing insights into the actions of conspecifics (Gallese *et al*. 1996; Rizzolatti *et al*. 1996, 2001; Umilta *et al*. 2001; Kohler *et al*. 2002). In recent years, evidence has accumulated for the existence of a mirror neuron system (MNS) for actions in humans (see figure 1*a*). Arguably, the most convincing evidence comes from functional magnetic resonance imaging (fMRI) and transcranial magnetic stimulation (TMS) studies. FMRI indirectly measures brain activity by estimating the level of blood oxygenation in cubes of brain tissue named voxels, whereas TMS uses magnetic stimulation to either stimulate or transiently impair a cortical region. FMRI shows that the ventral premotor cortex (BA44/6), inferior parietal lobule (IPL), as well as somatosensory areas (BA2 in particular) involved in executing the actions become reactivated while subjects view or hear similar actions performed by others (Grezes *et al*. 2003; Buccino *et al*. 2004; Gazzola *et al*. 2006, 2007*a*). Finding the same voxel involved in execution and perception, however, cannot ensure that the same neurons within the voxel (which is usually around 3 × 3 × 3 mm in size) are involved in both cases (Dinstein *et al*. 2008). Various researchers are now taking on the challenge to create experimental fMRI designs that can better address the neural response selectivity in the MNS than the usual movement observation and imitation protocols (Dinstein *et al*. 2007). In contrast, TMS experiments show that observing the actions of others specifically facilitates the execution of similar actions (Fadiga *et al*. 1995) and that applying repetitive TMS on the premotor or somatosensory cortex impairs this motor facilitation (Avenanti *et al*. 2007; Catmur *et al*. 2009). This demonstrates both that the vision of an action directly activates motor programmes for executing similar actions and that the link between vision and action occurs in the somatosensory and premotor areas identified by the fMRI experiments. This suggests that the MNS is indeed where perception meets action in the brain.

The possible roles of the MNS are still a matter of debate. Many would agree that mirror neurons are well positioned to support understanding *what* action another individual is performing and *how* it is being performed (Thioux *et al*. 2008). Anyone witnessing a person's hand reach for an orange on a table will instantaneously recognize the goal of the action: reaching to grasp. This mirroring seems to occur primarily at the level of motor goals. For example, aplasic subjects born without hands and arms activate foot and mouth representations when they observe hand actions they would perform with these effectors (Gazzola *et al*. 2007*b*). In addition, the disruption of putative mirror neuron areas in humans either through lesions (Pazzaglia *et al*. 2008*a*,*b*) or repetitive TMS (Pobric & Hamilton 2006) impairs the capacity of subjects to process the actions of others (see, however, Bell 1994; Halsband *et al*. 2001; Moro *et al*. 2008). A second important function attributed to the MNS in humans is imitation. This idea is supported by observations that a portion of the MNS (in pars opercularis of the inferior frontal gyrus (IFG)) is more active during imitation than the sum of activity during execution and observation (Iacoboni *et al*. 1999; see Catmur *et al*. 2009, for a discussion). A third function attributed to the MNS is that of empathizing with others, based on the finding that subjects that score higher on a questionnaire measuring their tendency to place themselves in the other person's shoes activate their MNS more strongly while hearing the actions of others (Gazzola *et al*. 2006).

### (b) Sharing sensations in the somatosensory cortex

In addition to a shared circuit for actions in the premotor and parietal cortex, there is evidence for a shared circuit in the somatosensory cortex that maps the perception and experience of tactile sensations. Keysers *et al*. (2004) showed their subjects movies of someone else's legs being touched with a stick. The same subjects were later touched on their own legs to localize their primary and secondary somatosensory cortices (SI/SII, see figure 1*b*). Part of SII that was active while the subjects felt touch on their own body became reactivated while viewing someone else being touched in similar ways. Activations in SI during touch observation were present, but were substantially weaker. Blakemore *et al*. (2005) found that the observation of touch was associated with an activity in SI that was somatotopically organized: different regions of SI react to the observation of someone being touched on the neck and the face. In the same experiment, the authors found that a subject with vision-touch synaesthesia, who reported vivid tactile QUALIA while seeing the tactile sensations of others, had increased activity in SI during the vision of touch. Electroencephalography (EEG) studies allow for a temporally precise measurement of components of electrical activity in the brain. Recently, an elegant EEG study by Bufalari *et al.* (2007) showed that in non-synaesthetes electrical activity in response to stimulation of the hand can be modulated by the sight of someone else being touched. The latency of this component (45 ms) suggests that within the mosaic of brain areas composing SI, those receiving direct thalamic input (BA3) are only active while experiencing touch on one's own body. The second stage of processing (BA1/2) is modulated by perceiving other people's experiences: stronger activity in these regions predicts how intense observers judge the sensations of others. Together, these studies suggest that under normal circumstances the earliest stages of cortical somatosensory processing (BA3) remain private, that is reserved for our own tactile sensations, while later stages (BA1/2 and SII) can serve to vicariously share the tactile sensations of others. Individuals with synaesthesia show that if the earliest stages are activated more strongly, the observer will experience touching of others literally as if being touched themselves. Similarly, while the MNS codes for both the execution and the perception of actions, the primary motor cortex (MI) is usually not active while viewing the actions of others (Gazzola & Keysers 2009). The absence of vicarious MI activity is quite natural as observers do not normally move while viewing the actions of others. The existence of patients that cannot refrain from overtly imitating behaviours they observe (Lhermitte *et al*. 1986) suggests that active inhibition is responsible for blocking the outflow of activity from premotor regions to MI (Preston & de Waal 2002; Gazzola & Keysers 2009). These mechanisms could help us distinguish our own actions and sensations from those of others which are shared in our (pre)motor and somatosensory regions.

In summary, primates readily activate premotor and parietal cortical areas involved in action execution when they see someone perform a goal-directed action. This simulation might be useful for understanding the action and its goal, and enable a more or less automatic imitation of someone's actions. There is mounting evidence to suggest that a similar neural mechanism involved in action imitation may also apply to the domain of sensations. Recently, it has been proposed that beyond actions and tactile perceptions our brain also readily simulates the emotions of others (Decety & Jackson 2004; Keysers & Gazzola 2006; Niedenthal 2007). Although this phenomenon seems superficially different from motor imitation, similarity in the neural processes suggests they are deeply related. This review examines the evidence for the presence of mirror mechanisms in sharing the emotions of other individuals.

## 2. Affective sharing of emotions

One of the key challenges in studying the sharing of emotions using neuroscientific methods is being able to trigger the emotion. Although this is generally difficult, there are some exceptions such as disgust and pain. Therefore, we will focus on the affective sharing of these particular emotions in the following section. In the case of pain, we will illustrate that emotion simulation not only involves an affective component (i.e. concerning sensations of pleasure and displeasure), but also a motor and sensory component, which will be addressed in the subsequent section. The interaction between the components is largely unknown but, as we will see in a subsequent section, recent experimental evidence suggests one potential route of communication from motor to affective mirror systems. The term mirror system implies that there is a certain degree of specificity: we map what we see onto our own neural substrates for that specific action, sensation or emotion. We will use the emotion fear to show that there is little evidence for a consistent mapping of particular emotions onto particular brain regions. Instead, different networks seem to be involved dependent on the process by which the emotion is accessed. In addition, the activation strengths of the different components are likely to be related to the quality of the emotion and its associated output. In the concluding comments, we will discuss the various functions that emotion simulation could subserve through its integration with, and modulation by, higher cognitive functions.

### (a) Sharing of disgust

**Figure 2.**
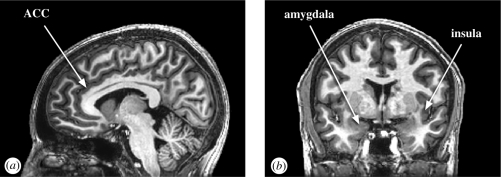
Anatomical locations of affective components of simulation. (*a*) Sagittal view of a human brain with the location of the anterior cingulate cortex (ACC). (*b*) Coronal view of a human brain showing the location of the insula and the amygdala.

Disgust is closely related to the phylogenetically primitive sensation of distaste. In its most basic form, from which more developed forms such as moral disgust may have evolved, it involves an oral defence to potentially harmful foods and body products (Haidt *et al*. 1997; Rozin *et al*. 2000). This makes disgust relatively easy to trigger repeatedly using aversive tastes and odours. The primary experience of taste and distaste can be located in the transition zone between the anterior part of the insular cortex together with the frontal opercular taste cortex (Yaxley *et al*. 1990; Small *et al*. 1999), a region we refer to as the IFO. The experience of unpleasant odours triggers activity in a similar region (Royet *et al*. 2003). Through its numerous connections to structures such as the orbitofrontal cortex (OFC), frontal operculum, anterior cingulate cortex (ACC), lateral premotor cortex, basal ganglia, temporal lobe and amygdala, the insula (see figure 2*b*) can anatomically fulfil the requirements for associating offensive tastes and smells with other people's expressions of disgust (Augustine 1996). This is supported by the finding of distinct electrophysiological responses in the anterior insula (AI) to facial expressions of disgust in the observer (Krolak-Salmon *et al*. 2003).

A seminal study in 2003 established a functional codependence of disgust experience and perception on the IFO. To reliably induce disgust, Wicker *et al*. (2003) puffed unpleasant odours in a mask placed over the subject's nose and mouth. When brain activations were compared between this condition and one in which subjects only viewed movies of an actor expressing disgust after sniffing the content of a glass, they demonstrated an overlap in the left IFO between the perception and experience of disgust. This result was later confirmed by showing that the experience of unpleasant tastes also overlaps in the IFO with the observation of others’ facial expressions of disgust (Jabbi *et al*. 2007).

Interestingly, the IFO not only seems to be *recruited* while viewing and experiencing disgust, but it also seems *essential* both for the first- and third-person perspective of disgust. Two patients with lesions encompassing the anterior insular activations found above are unable to feel disgust and are impaired in recognizing this emotion in other individuals (Calder *et al*. 2000*b*; Adolphs *et al*. 2003). This is consistent with a large lesion study showing that the somatosensory cortex/anterior supramarginal gyrus and surrounding insular region are essential for recognizing emotions from visually presented facial expressions (Adolphs *et al*. 2000). In addition, the AI is implicated in disorders associated with an impaired ability to recognize disgust such as obsessive-compulsive disorder (Breiter *et al*. 1996; Sprengelmeyer *et al*. 1997; Calder *et al*. 2001), Wilson's disease (Wang *et al*. 2003) and Huntington's disease (Sprengelmeyer *et al*. 1996; Gray *et al*. 1997; Wang *et al*. 2003; Hennenlotter *et al*. 2004).

The role of the IFO goes beyond the perception and experience of disgust. The fact that electrical stimulation of the anterior sector of the insula evokes nausea and visceromotor activity (Penfield & Faulk 1955; Calder *et al*. 2000*b*) demonstrates its role in controlling visceral sensations and related autonomic responses. The IFO not only instantiates the representation of bodily states, but it also makes these representations consciously available as subjective feeling states. More activity and grey matter in the insula for instance predicts that people are better at judging their visceral bodily states (Critchley *et al*. 2004). In addition, the strength of activation in the IFO when witnessing expressions of disgust is stronger in individuals that report experiencing more distress while witnessing the distress of others (Jabbi *et al*. 2007). This suggests that the IFO is involved in the involuntary sharing of emotional states often referred to as emotional contagion (Hatfield *et al*. 1993). In sum, this indicates that the IFO might have a dual function: it can translate the observation of other people's facial expressions into similar visceral states of the self (Critchley *et al*. 2005) and make these states consciously available for sensing the emotional state of other people (Keysers & Gazzola 2007).

### (b) Sharing of pain

Although pain is not traditionally considered a basic emotion, it is a strong feeling state that can, akin to disgust, be triggered repeatedly and reliably in a research environment. Neuroimaging studies show involvement of the dorsal ACC and AI in processing the unpleasantness of physical pain (for a review see Peyron *et al*. 2000). In the ACC (see figure 2*a*), nociceptive-specific neurons are found which respond to contralateral noxious thermal and/or mechanical stimulation, but not to their non-painful equivalent (Hutchison *et al*. 1999; Vogt 2005), which is consistent with the ACC's role in pain experience. Importantly, Hutchison *et al*. (1999) also demonstrated the existence of single neurons in the ACC which are active during both the sensation and perception of pain. This suggests that, similar to touch and disgust, pain is a feeling that we simulate.

In a seminal study, Singer *et al*. (2004) tested romantic couples in a situation where one was lying in the scanner and was informed by a symbol on the computer screen when her lover was receiving a painful stimulation. Knowing that her lover was in pain activated parts of the pain matrix that were also active when a noxious stimulus was applied to the subject herself: the AI and ACC. Numerous other studies (reviewed in Singer & Lamm 2009) found activation of the insula and the ACC associated with the observation of stimuli depicting pain-inducing events. For instance, static images of a knife cutting a hand, or a foot stuck in the door (Jackson *et al*. 2005), and videos of needles being inserted into human body parts (Cheng *et al*. 2007) all activate these areas. Even in the absence of a direct pain-inducing event, the observation of a facial expression of pain activates the ACC and AI. This was shown for the observation of dynamic facial expressions of moderate pain compared with neutral expressions (Botvinick *et al*. 2005), and when comparing painful expressions with angry ones (Simon *et al*. 2006). In addition, these regions respond more strongly to intense than mild facial expressions of pain (Saarela *et al*. 2007). Akin to disgust, pain simulation in affective centres (ACC, AI) is correlated with interindividual differences in empathy (Singer *et al*. 2004). This led some researchers to emphasize the representations of the other person's subjective unpleasantness in understanding someone else's pain (Singer *et al*. 2004, 2006; de Vignemont & Singer 2006). Recent studies suggest, however, that sensory and motor components may also play a role.

## 3. Sensory and motor components of emotion simulation

Pain is often characterized by a motor response (e.g. facial expression of pain) and frequently has a clear sensory component (e.g. a needle entering the skin), which resembles other emotions. In fact, the pain matrix, which designates the collection of areas involved in the experience of pain, consists of a somatic/sensory as well as an affective/motivational component (Jackson *et al*. 2006). Brain areas that are involved in representing the sensory aspect of physiological pain are the thalamus, SI/SII and the (posterior) insular cortex (Peyron *et al*. 2000; Price 2000). Activation of the motor cortex and the cerebellum is also reported in studies of pain experience (Peyron *et al*. 2000). The next section reviews evidence of motor and somatosensory simulations during pain perception in particular and emotion perception in general, suggesting there is more than merely affective simulation.

### (a) The case of pain

In some cases, people might share not only affective but also motor and somatosensory representations with other people in pain. In the EEG study cited earlier, Bufalari *et al.* (2007) show that the degree to which the sight of other people's tactile and nociceptive sensations modulates neural activity from the crown of SI (BA1 and 2) depends on the rated stimulus intensity. This suggests that sensory components of the pain matrix can be activated by the vision of other people's pain. At first glance, fMRI studies seem more ambiguous about the role of SI and SII in pain perception. As far as the sensory cortex is concerned, about half of the studies found activity in SI/SII (e.g. Lamm *et al*. 2007; Moriguchi *et al*. 2007; Decety *et al*. 2008) and half did not (e.g. Singer *et al*. 2004, 2006; Jackson *et al*. 2005). At least three factors could explain these differences. First, vicarious SI/SII activity might be of modest intensity and the sensitivity of the method and sample size of the experiment then determine whether it is significant. For example, Jackson *et al*. (2005) failed to find SI/SII activation in their fMRI study; Cheng *et al.* (2008), using the same stimuli with hands and feet in painful situations, demonstrate suppression of the mu rhythm at the postcentral gyrus using magnetoencephalography (MEG), a technique that measures electrical activity in the brain by use of magnetic fields. The mu rhythm is suppressed during both the execution and performance of actions and is for that reason seen as an indicator of mirror neuron activity (Pineda 2005). Also, Jackson *et al*. (2005) fail in their sample of 15 subjects to find the SI/SII activity that Moriguchi *et al*. (2007) did find in their group of 30 subjects using the same stimuli. Second, the type of comparison may also play a role. Singer *et al*. (2004, 2006) subtracted conditions in which subjects receive an electric shock above the *tactile* threshold from one above the *pain* threshold. Likewise Saarela *et al*. (2007) subtract mild facial expressions of pain from more intense ones. By subtracting one tactile condition from another, somatosensory activation present in both conditions may be lost. Indeed Cheng *et al*. (2007) find somatosensory activation both when subjects view needles (pain) being inserted into different body parts and when these body parts are touched by a q-tip (no pain). Moreover, activation of the somatosensory regions disappears when pain scenarios are contrasted with the neutral ones. Although somatosensory regions do not survive in a whole-brain analysis, Cheng *et al*. (2007) show that S1 activation correlates with pain intensity. In addition, region of interest (ROI) analysis on the left postcentral region (functionally defined by the somatosensory signal change during the pain condition) shows that watching painful situations results in stronger activation than watching the neutral equivalents. Again, when using more sensitive methods, somatosensory involvement during pain perception is demonstrated. A third factor explaining the contradictory findings concerning the involvement of the sensory cortex in pain perception could be the experimental design used: some neuroimaging studies draw more attention to general unpleasantness instead of focusing on a specific body part. For example, Singer *et al*. (2004) did not find an overlap in somatosensory cortices for perception of pain in self and a loved one. However, pain in the other was indicated by a cue and no pain-related behaviour was visible. Similarly, studies using facial expressions of pain do not localize the source of the pain on the body. This could be the reason they often fail to find somatosensory activations in response to pain (Botvinick *et al*. 2005). While putting more emphasis on the affective side of pain reduces somatosensory engagement, evaluating the sensory consequences of pain conversely leads to increased activity in somatosensory areas (Lamm *et al*. 2007).

Evidence that simulation of pain can involve the motor system as well comes from Avenanti *et al*. (2005). They found that during the observation of pain applied to hands, motor excitability (as measured using TMS-induced motor evoked potentials, MEP) in the corresponding hand muscles of the observer is decreased. In addition, the amplitude of MEP inhibition correlates with sensory aspects such as pain intensity. Along the same lines, an fMRI study contrasting pictures of faces displaying pain varying from high to low intensity found that various nodes of the motor system (BA45, SMA, BA6 and left IPL) were sensitive to intensity differences in displayed pain (Saarela *et al*. 2007). The role of motor activation could be twofold, with the MNS registering the actions of the face and body, while the supplementary motor area (SMA) could be involved in programming defensive movements during pain perception (Decety *et al*. 2008). These studies indicate that the pain of others is represented in a mosaic of brain regions involving affective, somatosensory and motor representations, but the precise factors determining the relative importance of these various nodes remain to be elucidated.

### (b) Other emotions

The study of pain demonstrates that sharing the emotions of others may not be limited to sharing their affective states: motor and somatosensory aspects of emotions may also be shared. In most cases, we deduce the emotional states of others from their motor behaviour: we know people are happy because they smile when they are happy, and we know when people are disgusted because they turn up their noses. Could a system similar to the mirror system for goal-directed actions allow an observer to share the facial and bodily emotional expressions of others?

The repertoire of the motor mirror system indeed extends from hand actions to a wide range of body actions including facial actions (e.g. Buccino *et al*. 2001). In monkeys, mirror neurons were documented that react to the observation of specific mouth actions: some ingestive mouth actions such as sucking, but also, and most interestingly, some communicative ones such as lip smacking (Ferrari *et al*. 2003). Several brain-imaging experiments in humans also suggest that we activate our premotor cortex upon viewing an emotional facial expression. Activity in the pars opercularis of the IFG and the ventral premotor cortex (vPMC) was reported in several brain-imaging studies in which subjects observed emotional facial expressions. Interestingly, premotor activity is found for the observation of both dynamic (Wicker *et al*. 2003; Hennenlotter *et al*. 2005; van der Gaag *et al*. 2007) and static stimuli (Carr *et al*. 2003; Wild *et al*. 2003; Leslie *et al*. 2004). Additional evidence for the role of the motor cortex in emotion perception comes from two studies showing that viewing an emotional facial expression interferes with a simple facial motor task, which translates into an increase of activity in the vPMC/IFG that is correlated with the intensity of the emotion (Wild *et al*. 2003; Lee *et al*. 2008). Additionally, the amount of facial movement during the imitation of emotional expressions correlates with activity in the MNS (Lee *et al*. 2006).

The somatosensory cortex together with the ventral premotor cortex and IFG seem to be recruited when perceiving mouth actions (Gazzola *et al*. 2006) and natural facial emotional expressions (Wicker *et al*. 2003; Winston *et al*. 2003; Hennenlotter *et al*. 2005). Expressions causing the most somatosensory activity during execution also caused the most activity during observation (van der Gaag *et al*. 2007). In line with pain studies, activity of the somatosensory cortex is not reported consistently across studies. This might be explained by sensitivity of the measurement, the type of stimulus (i.e. static versus dynamic), a reporting bias (e.g. report of only the peak coordinates of an active cluster), or the type of comparison (i.e. against baseline or a neutral condition). Importantly, a large lesion study has shown that lesions to the right somatosensory cortices (centred on the most ventral part of the somatosensory cortex, where the face is represented) impair the ability to recognize emotions from visually presented faces (Adolphs *et al*. 2000). Apparently, activation of somatosensory representations of the face when viewing emotions is crucial for emotion recognition.

In summary, we may get access to the facial state of another person (i.e. the configuration of facial muscle groups) by reproducing in our premotor cortices the contractions of the muscles we observe, and by feeling the effect of these (simulated) contractions in our own somatosensory cortices. This idea is strongly supported by observations that even subliminal exposure to emotional facial expressions triggers measurable movements of the observer's facial musculature that resemble those observed. This phenomenon is called facial mimicry (Dimberg *et al*. 2000). Whether such sensorimotor simulation could be important for generating a model of the affective state of others will be discussed in the following section.

## 4. From motor to affective simulation

Psychological theories have linked overt facial mimicry (as measured by an electro-myograph or through observation) to emotional contagion and emotion understanding (James 1884; Lipps 1907; Niedenthal 2007). Given that our brain has a lifelong experience with the correlation between our own facial configuration and our personal internal affective states, the simulation of other people's facial configuration could trigger matching affective states. Intriguingly, there is only limited evidence that the amount of facial mimicry correlates with the amount of emotional contagion and/or understanding. While Niedenthal *et al*. (2001) show that blocking facial mimicry leads to slower detection of facial expression change, Hess & Blairy (2001) could not demonstrate a direct link between degree of facial mimicry and accuracy of emotional recognition. Additionally, studies in disorders affecting facial expressivity such as Möbius syndrome and facial paralysis show no striking emotion recognition impairment (Calder *et al*. 2000*a*; Keillor *et al*. 2002). While it is difficult to directly translate the concepts of facial mimicry and emotional contagion into testable neural hypotheses, it seems likely that if facial mimicry were to trigger emotional contagion, areas such as the primary somatosensory or motor cortex, known to directly sense or cause facial movements, would be most strongly connected to the insula, which is thought to represent a neural correlate of emotional contagion. To explore this possibility, Jabbi & Keysers (2008) performed a functional connectivity study using Granger causality. A functional connectivity study can identify brain regions whose connectivity (i.e. correlation in activity) is modulated by the task. A correlation between two brain regions implies a connection, but does not necessarily indicate causation. Granger causality can, however, determine whether a time series A is useful in forecasting another time series B more than B is able to predict A. Jabbi & Keysers (2008) used the region of the IFO that is common to the experience and observation of disgust as a seed or reference region in their study. They found that activity in the IFO is Granger-caused by activity in the region of the IFG that is active both while observing and generating facial expressions. In contrast, there was no enhanced effective connectivity with the somatosensory cortex or the primary motor cortex. This may explain why the IFG is not just responsive to facial movement, but is more active when attention is drawn to emotional or socially relevant properties (Gur *et al*. 2002; Lawrence *et al*. 2006; Scheuerecker *et al*. 2007; Schilbach *et al*. 2007). It also suggests that the link between motor simulation and emotional contagion may not be through overt facial mimicry, as suggested by early psychological theories (Lipps 1907), but instead through a covert simulation in high-level motor regions (Carr *et al*. 2003). This may help clarify why motor simulation can be important in emotion understanding even in the absence of a tight correlation between overt facial mimicry and emotional contagion. Future studies are needed to further investigate this provocative hypothesis.

In summary, regions involved in simulating facial expressions indeed seem to trigger an affective simulation of the hidden inner states of others. In this process, the link between our own (visible) facial expression and (invisible) internal states could serve as a Rosetta stone to derive hidden internal states from the observable actions of others. It is likely that bodily expressions of emotions (such as body postures) could be processed in similar ways (de Gelder *et al*. 2004). There are, however, likely to be many routes to equally many types of emotions which are processed in various regions of the brain.

## 5. The specificity of sharing emotions

An important theme in the neural study of emotion has been the search for brain areas that are selectively involved in particular emotions. Many for instance associate the ACC with the emotion of pain (Hutchison *et al*. 1999), the IFO with the emotion of disgust (Calder *et al*. 2001; Adolphs *et al*. 2003) and the amygdala with the emotion of fear (Adolphs *et al*. 1994). Such an organization would be a powerful instrument to examine the degree to which the observation of a particular emotion in *others* is translated into representations of a similar emotion in the *self*. Unfortunately, most studies do not lend themselves well to answering this question: some lump together the observation of various facial expressions in the design of the experiment (e.g. Carr *et al*. 2003; Leslie *et al*. 2004), while others focus on only one emotion (e.g. Botvinick *et al*. 2005; Hennenlotter *et al*. 2005; Grosbras & Paus 2006). Additionally, many other studies do not use a condition where the subjects experience the emotion themselves (e.g. no studies have been performed yet combining perception and experience of fear). Overall, the available data shed increasing doubts on the existence of a reliable mapping of particular emotions onto particular brain regions.

### (a) The case of fear

Fear is probably the most widely studied basic emotion, and much interest in the literature on fear has focused on the amygdala (see figure 2*b*). This structure is often thought to be involved in processing facial, vocal and bodily signals of fear as well as in the experience of this emotion and fear conditioning (e.g. Halgren *et al*. 1978; Phan *et al*. 2002; LeDoux 2003; de Gelder *et al*. 2004). The fact that certain patients with lesions in the amygdala seem to show deficits in the recognition and experience of fear (e.g. patient SM: Adolphs *et al*. 1994; Tranel *et al*. 2006; patient YW: Broks *et al*. 1998; patient NM: Sprengelmeyer *et al*. 1999) but not of other basic emotions has led some to propose that the brain simulates the fear of others by activating states of fear in a fear-selective amygdala (Goldman & Sripada 2005). However, recent neuroimaging and patient studies challenge this view. First, it is unclear whether patients with lesions in the amygdala are unable to experience fear: two-week-old primates with amygdala lesions display *more* fear (more grimaces and screams) during social interactions than non-lesioned conspecifics (Amaral *et al*. 2003); Anderson & Phelps (2002) report an amygdala-lesioned patient who expresses a normal range of emotion; and even the most widely tested lesion patient SM displays a normal range of affect and emotion during social interaction (Tranel *et al*. 2006). Second, it is unclear whether lesions in the amygdala *directly* impair the recognition of fear: half the reported patients with amygdala lesions display normal fear recognition (see Keysers & Gazzola 2006); SM is unimpaired in recognizing fear from vocal and bodily expressions of fear (Adolphs & Tranel 1999; Atkinson *et al*. 2007) and if instructed to look at the eyes of people (which she does not spontaneously do), even her recognition of fearful facial expressions is normal (Adolphs *et al*. 2005). The role of the amygdala in recognizing fear may have less to do with the actual recognition, but more with directing attention to the salient parts of the environment (e.g. eyes) through its connections with high-level visual areas (Vuilleumier *et al*. 2004), and then the actual recognition occurs elsewhere. Indeed, an increasing number of fMRI studies find that the amygdala is similarly recruited by movies of positive and negative facial emotions (Kilts *et al*. 2003; van der Gaag *et al*. 2007), except in fearful individuals (Ewbank *et al*. 2009) or after administration of norepinephrine and cortisol to simulate stress (Kukolja *et al*. 2008). When all these results are taken together, they suggest that the role of the amygdala in experiencing and recognizing fear is more indirect than previously suggested, which sheds doubt on the proposal that this structure embodies a selective simulation of fear.

### (b) Other emotions

Similar problems apply to other brain regions that have been considered relatively selective for particular emotions. Van der Gaag *et al*. (2007) systematically compared the observation of movies of happy, fearful, disgusted and neutral facial expressions. They could not find fear selectivity in the amygdala. Furthermore, disgust *and* the other emotions activated the IFO without significant differences between any of the emotions. The fact that the IFO seems similarly important for the simulation of pain and disgust (see above), also makes the lack of specificity of this structure apparent. Moreover, this structure is not only recruited by both pain and disgust, but it is activated more strongly in more empathic individuals for both of the emotions. Furthermore, interindividual differences in empathy also explain activity in the very same voxels during the observation of positive facial expressions (Jabbi *et al*. 2007) with no difference between emotions. Therefore, the insula does not seem to be a centre for disgust. One alternative hypothesis is that the insula may play a broader role in emotion processing by translating what we perceive into visceral responses that colour our subjective feelings (Craig 2002). Since disgust is related to visceral responses in particular (retching, nausea), we may in some cases rely more strongly on the insula for recognizing that particular emotion in ourselves and others. Finally, even the link between the cingulate cortex and a particular emotion such as pain may be an oversimplification. Although neurons responding to painful stimuli exist in the ACC, Vogt (2005) suggests that many regions of the cingulate cortex are not specific for a particular emotion but for a particular output. For instance, viewing sad faces is associated with increased activity in the subgenual ACC because of the role of this region in autonomic integration, while the perception of pain and fear causes overlapping activations in the anterior midcingulate cortex (aMCC) because of its strong motor connections, which prepare the body to react to these challenges (Vogt 2005).

In summary, although there is vast evidence for a role of the amygdala in fear, a role of the AI in disgust and a role of the ACC in pain, these activations are not emotion specific. Signals of fear could enhance amygdala activity (in particular in stressful situations) because they indicate a potential threat and as a result visual attention to the outside world is increased. Similarly, visceral responses mediated by the AI are probably more important for disgust and the aMCC with its strong motor connections might be particularly relevant for pain. This might explain why activity in the amygdala is often found for fear, activity in the AI is often found for disgust and activity in the ACC is often found for pain. However, activity in these regions is unlikely to be directly linked to a particular emotion and for this reason simulation of a particular emotion is also unlikely to be related to a particular brain region.

A key challenge for the field of emotion in general, and the simulation of emotions in particular, will be to examine whether individual neurons—within brain regions that are not specific as a whole—may represent some emotions more than others both during self-perception and other-perception. In the case of pain, for instance, there seems to be a rostro-caudal functional organization of ACC and IC with self-perception involving more caudal areas than other-perception (Jackson *et al*. 2006; Morrison & Downing 2007). Applying methods such as (cross-modal) adaptation (Dinstein *et al*. 2007), which are used in the study of motor actions, might help address the question of neural specificity in the emotional domain.

We should, however, be wary of treating brain regions as separate entities. Simulation is a highly integrated process which is likely to depend on the networks connecting various regions. Indeed, much of the distinction between self and other during social interactions may depend on differences in the networks in which shared circuits are engaged. For example, although the IFO is active when observing, feeling and even imagining disgust, effective connectivity analysis shows that the involved networks are quite different (Jabbi *et al*. 2008): during experience, the IFO is embedded in a network composed of somatosensory, gustatory/motivational and motor output regions; during mental imagery (triggered by written scripts) in a network of language processing, semantic memory (temporal pole) and mental imagery (SMA) areas; finally, during observation, the IFO receives its strongest emotional input from the right BA45, which is involved in execution, observation and imitation of facial expressions. The same is true for pain: the ACC and AI are involved in both the experience and the observation of pain, but the functional network during self-perception is different from the network that is activated during the perception of others in pain (Zaki *et al*. 2007).

## 6. Concluding comments

### (a) Role of simulation

Neuroimaging experiments show that we activate common circuits when observing sensations or emotions felt by others, and when experiencing these sensations and emotions ourselves. This clearly suggests that seeing someone else experiencing touch, disgust or pain triggers much more in us than a purely theoretical, disembodied interpretation of other people's mental states. Witnessing someone experiencing an emotion or a sensation is associated with a pattern of activity in our brain embodying their actions, sensations and affective states. What could be the role of this automatic cortical simulation?

The motor component of simulating other people's facial expressions can have two purposes. One is directly social and arises when the observer of a facial expression not only simulates the facial expressions of others, but allows this simulation to show on his/her face. Such facial mimicry facilitates social contacts and could increase the survival of individuals by increasing their social success (see Chartrand & Bargh 1999; van Baaren *et al*. 2009). The overt outflow of simulated facial expressions, however, depends on the social context: people refrain from imitating people's smiles if they are in competitive contexts or deal with an outgroup member (see Lanzetta & Englis 1989; van Baaren *et al*. 2009); motor simulation of goal-directed actions can be overt during imitation but remains covert in most situations. The second function of motor simulation seems to be a way of bridging the observable behaviour of others with hidden internal states that correspond to these behaviours. It could do this by triggering a simulation of affective states through the connections of premotor regions with the IFO (Jabbi & Keysers 2008). This circuitry does not require the primary motor cortex and therefore does not require the motor simulation to become overt. As was previously discussed, this could explain why the amount of overt facial mimicry does not directly predict how accurate observers are at judging the emotions of others, or how much they are affected emotionally by the emotions of others (Gump & Kulik 1997; Blairy *et al*. 1999; Hess *et al*. 1999; however, see Niedenthal *et al*. 2001; Sonnby-Borgstrom 2002). The importance of such motor simulation for feeling what goes on in others derives from lesion studies that show that lesions in these regions impair the recognition of affect in others (Adolphs *et al*. 2000). The affective simulation that can be triggered by the motor simulation of others’ behaviour and/or by mental imagery of their states derived from other sources of information (Jabbi *et al*. 2008) is likely to have a dual function as well. On the one hand, it probably allows us to feel what goes on in others: lesions in regions involved in sensory (SI/SII + posterior insula) and/or emotional (IFO) simulation indeed impair an individual's capacity to judge the emotions of others (Adolphs *et al*. 2000, 2003; Calder *et al*. 2000*b*). On the other hand, beyond providing a direct understanding of the emotions felt by others and allowing the selection of appropriate behavioural responses, affective simulation may help ‘synchronize’ the emotional states of members of a group.

The study of the neural basis of simulation makes a further functional prediction. Given that emotions are shared through a mosaic of motor, somatosensory and affective simulations, people's reactions to other people's emotions may be expected to differ in fine-grained ways. For instance, certain people could engage in more motor and less affective simulation. Others may have the reverse relationship. The psychological literature indeed supports the idea that empathy has multiple separable subcomponents, which is in contrast to the layman's vision of empathy as a unitary system. Many separate cognitive empathy from affective empathy (Mehrabian & Epstein 1972; Davis 1983; Baron-Cohen *et al*. 2001) while others additionally distinguish motor empathy (Blair 2005). Finally, even affective empathy can be further divided into personal distress (the contagious sharing of others’ distress) and emotional concern (the wish to help that is triggered by the distress of others), with these forms developing at different ages (Preston & de Waal 2002). Although the distinctions made by neuroscientists and psychologists differ—the former being driven by neuroanatomical and the latter by functional considerations—recent evidence suggests that these distinctions may be linked. Affective forms of empathy correlate with brain activity in affective brain regions (IFO while witnessing the disgust or pleasure of others: Jabbi *et al*. 2007; and while sharing the pain with a loved one: Singer *et al*. 2004). The less affective forms (cognitive perspective taking), however, correlate with the activity in non-affective brain regions (premotor and somatosensory areas during action observation: Gazzola *et al*. 2006; Avenanti *et al*. 2009). Some psychologists would rather not label personal distress as a form of affective empathy because it involves a self-oriented rather than an other-oriented affective response to the emotions of others (Batson *et al*. 1987; Eisenberg 2006). In fact, there are studies showing that the more a person attributes their own traits to another person and the higher the person's own distress to discomfort in others, the less strong the empathic responses are in motor and somatosensory regions (Lawrence *et al*. 2006; Avenanti *et al*. 2009). Notwithstanding the debate about whether personal distress should be labelled as a form of empathy or not, it is still largely unclear why individuals differ in the composition of their empathy to begin with and how such differences could be influenced by training.

Finally, the automatic sharing of both affect and action with others may have a very fundamental role for learning. While it remains unclear whether the MNS involved in actions may be partially inborn, it certainly is plastic. For instance, the training involved in becoming a dancer or pianist increases the MNS response to perceiving others perform that particular dance (Calvo-Merino *et al*. 2005; Lahav *et al*. 2007), and practice can virtually reverse the behaviour of the mirror system (Catmur *et al*. 2007). It has been suggested that the association between performing an action and perceiving oneself perform the action may form the basis for this plasticity (see Heyes 2001; Keysers & Perrett 2004; Catmur *et al*. 2009). For actions that we do not see ourselves perform (e.g. facial expressions), experience of early imitation by parents may be the key to learning (del Giudice *et al*. 2009). While motor simulation alone has often been taken as the neural basis of learning by observation, this explanation falls short of explaining how observers can learn which of the actions of others are worth learning. This problem might be naturally solved by the brain using a combination of affective and motor simulation. If viewing another individual perform action A resulted in a positive outcome, and action B resulted in a negative outcome, the brain of the observer would vicariously coactivate affective reward areas and motor representations of A, but coactivate pain areas together with representations of B. This would lead to assimilating behaviour A but not behaviour B through the very mechanisms of individual trial and error learning and operant conditioning.

### (b) Beyond simulation

A variety of authors have criticized simulation theory (ST) based on the fact that it cannot explain all facets of social cognition (e.g. Jacob & Jeannerod 2005; Saxe 2005; Gallagher 2007) and that we still fail to have conclusive evidence in humans that the exact same neurons are involved in action perception and execution (Dinstein *et al*. 2008). As previously discussed, this criticism also applies to the case of emotions and sensations. We view the first critique as an experimental challenge that should inspire researchers in the next decade. As for the second, we believe that it is fruitless to create a competition between simulation views versus more cognitively inspired ‘mentalizing’ approaches in current social neuroscience research. There is no doubt that in many instances we rely on our knowledge of the person or the situation to make inferences about the state of mind of the other. If a salesman of second-hand cars smiled broadly while bragging about the quality of a rusty old car, our simulation circuitry could make us share his enthusiasm, but semantic knowledge about second-hand car salesmen could lead to a different conclusion. There is ample evidence that what we know about someone else can influence the simulation mechanism. For instance, the perceived fairness of the observed individual in pain influences how much pain will be shared (Singer *et al*. 2006). Similarly, the gender of the observed individual can influence our neural response (Simon *et al*. 2006). In addition, the sensory part of the pain matrix is engaged more when the perceived pain reaction of another person matches how we would respond ourselves (Lamm *et al*. in press). Therefore, we believe the interesting question is *how* these two processes are integrated in the brain (Keysers & Gazzola 2007). Brass *et al*. (2009) for instance suggest that it is the *control* of shared representations by the temporo-parietal junction (TPJ) and medial prefrontal regions (e.g. by virtue of assigning agency and suppressing externally triggered response tendencies) and not the shared representations *per se* that pave the way to understanding others. Various authors have implicated these regions in mentalizing and determining agency (Mitchell *et al*. 2006; Saxe 2006). Brass *et al*. (2009) suggest these regions not only control automatic imitative response tendencies but also shared representations involved in higher order social cognition. In fact, Cheng *et al*. (2007) show that when acupuncture practitioners watch needles being inserted into someone's body parts they do not activate their own pain matrix (ACC, AI, PAG) as naive subjects do, but they activate medial and superior prefrontal cortices and the TPJ instead. Possibly due to their knowledge of acupuncture, experts cognitively inhibit affective simulation and reduce their vicarious experience of pain intensity and unpleasantness. Understanding the influence of higher-level cognitive representations on simulation will be one of the key challenges in the coming years, and will be essential to understanding how our species has adapted to a world in which simulation can sometimes be adaptive and sometimes not (e.g. when having to fight an enemy).

## Glossary

Simulation: A form of neural processing of social information that involves activating neural states during observation that match those that the observer experiences in a similar situation. An example of motor simulation would be activating a pattern of motor activity while watching other people's actions that corresponds to the pattern found when the observer performs the same actions (Etzel *et al*. 2008).
Embodied cognition: By embodied cognition we mean any form of cognitive processing that is performed in representation codes that are specific to the body (see Goldman & de Vignemont 2009). Such codes include the motor codes implemented in primary motor, premotor and supplementary motor cortices; the somatosensory codes implemented in primary and secondary somatosensory cortices as well as in the insula; the visceromotor representations implemented in the insula.
Qualia: The phenomenological aspects of feelings and sensations making up our conscious experience.
Emotional contagion: Simulation as we use it refers to a process, namely how the brain attaches meaning to other people's states by recruiting representations of similar states of the self. Emotional contagion refers to the effect that this can have on the observer's mood or emotions: the emotional state of an observer comes to resemble that of the observed individual, for instance an infant starting to cry upon hearing another baby crying (Hess & Blairy 2001).
Empathy: We use empathy in its broadest sense unless otherwise specified: the process through which we are sensitive to other people's inner states (affective states in particular) by placing ourselves into either a similar state (feeling sad while seeing a friend cry) or a compassionate state (having tender feelings for a person in pain). This broad definition includes emotional contagion as well as what has been termed empathy in the narrow sense, that is an other-focused congruent emotion (Batson *et al*. 1987; de Vignemont & Singer 2006).
Imitation: We use imitation in its broadest sense here: the generation of a behaviour that follows and matches an observed behaviour. This broad definition includes what has been termed emulation (generating a behaviour that achieves the same goal as the observed behaviour) and ‘true imitation’ (generating an otherwise unlikely movement that matches the one observed in its details). This term also includes both automatic imitation, as measured using interference paradigms (see Brass *et al*. 2009), and voluntary imitation, as measured using more explicit instructions to imitate.
Motor goals: The proximal purpose of an action. We speak of motor to specify the pragmatic nature of the goals as can be implemented in the motor system, as opposed to more abstract goals or intentions that would be implemented in non-motor brain regions. The motor goal of grasping a glass of water would for instance be to hold the glass. This particular goal can be achieved in a variety of ways (e.g. you could grasp it with your left or right hand or with your feet), which places it above detailed motor programmes in the motor hierarchy. It is however different from a more distal goal or intention of grasping ‘to drink’ or ‘to throw the content into my enemy's face’. It refers to the ‘what’ level of an action (Thioux *et al*. 2008).
